# Broadening Core Research Ethics Principles: Insights from Research Conducted with Black Communities

**DOI:** 10.1002/eahr.60011

**Published:** 2025-09-27

**Authors:** Johanne Jean‐Pierre, Tya Collins, Khandys Agnant, Alicia Boatswain‐Kyte, Cameron Herman, Tanya Mathews, Bukola Salami, Carl E. James

**Affiliations:** ^1^ Associate professor in the Department of Sociology at York University; ^2^ Assistant professor in the Faculty of Education at the University of Ottawa; ^3^ PhD student at McGill University in the Department of Sociology; ^4^ Assistant professor at the School of Social Work at McGill University; ^5^ Research associate in the Department of Sociology at the University of Toronto; ^6^ Postdoctoral research fellow in the School of Communication Sciences and Disorders at McGill University; ^7^ Professor in the Department of Medicine at the University of Calgary; ^8^ Jean Augustine Chair in Education, Community & Diaspora at York University and Advisor on Equity in the Faculty of Education

**Keywords:** anti‐Black racism, Black communities, community‐based research, health equity, institutional review board (IRB), biomedical research, public health research, research ethics

## Abstract

Drawing from a 2023 symposium panel that focused on conducting health equity research with Black communities, we propose to expand our interpretation of core research ethics principles. In light of a surge of research conducted in Black diasporic communities since the 2020 killing of George Floyd, the symposium sought to enhance the quality and impact of research involving Black Canadians. We contend that by broadening the interpretation and application of respect for persons, beneficence, and justice, researchers will conduct impactful and transformative research projects that foster health equity. We emphasize the importance of not limiting the core principle of respect for persons to individual participants but to extend it to communities throughout the research process. Furthermore, we suggest that researchers can deepen their commitment to the core principle of beneficence or concern for welfare and design relevant and empowering research projects through meaningful community involvement. We highlight that to further the implementation of the core principle of justice, scholars should adopt a human development approach and mobilize innovative outreach recruitment strategies to ensure that Black communities have the opportunity to participate in biomedical and public health research while also benefiting from the knowledge produced.

There are three core principles that inform research ethics committees when they review research protocols. These principles are respect for persons, concern for welfare or beneficence, and justice. In Canada, these principles are enshrined in the Canadian Tri‐Council Policy Statement: Ethical Conduct for Research with Humans*—*TCPS 2,^1^ which serves as the national framework guiding ethical research practices. These principles also align with similar frameworks, such as the National Institutes of Health^2^ (NIH) guidelines and global ethical standards like the Declaration of Helsinki.^3^ Across borders, these principles play a determinant role in the structure of research protocols and the content of research methods curricula. These core principles have not always existed and tend to evolve as ethical concerns emerge. This article aims to broaden our interpretation and application of core research ethics principles to further health equity, drawing from insights shared at an interdisciplinary symposium entitled “Designing a Flourishing Future and Researching with Black Communities in Canada” held in Toronto, Canada at York University in November of 2023.

We observed a surge in research endeavors concerning race and Black communities following the deaths during police intervention of Black Canadians D'Andre Campbell in April of 2020 and Regis Korchinski‐Paquet in May of 2020, which occurred in temporal proximity to the death of George Floyd in May of 2020 in the United States. This symposium aimed to consolidate and expand the state of knowledge on conducting ethical research with Black populations. The panel discussed in this article convened health experts working with Black communities across various regions of Canada, employing diverse methodological approaches to offer insights and guidance on conducting health research that promotes equity and community development. This discussion featured contributions from panelists and coauthors of this article, Cameron Herman, Bukola Salami, Alicia Boatswain‐Kyte, and Tanya Matthews, whose research interests span food insecurity, nursing and public health, clinical social work and health disparities, and speech pathology studies, respectively. The panelists’ contributions constitute the foundation of this article as they expand our understandings of core research ethics principles through concrete examples derived from their own prior and ongoing research projects. The panel proceedings provide an occasion to interpret and apply core research ethics principles in ways that promote equitable practices during the design, conduct, and dissemination of health research conducted across various Black communities. While the examples discussed are context‐specific, the expanded interpretation of core research ethics principles that we propose can be adapted across various national and regional contexts. Prior to delving into their insights as they relate to the core principles of ethical research, we provide a brief overview of the historical harms inflicted on historically marginalized communities and emphasize the need for heightened attention and ongoing reflection on ethical research practices.

## HISTORICAL LEGACY OF UNETHICAL RESEARCH AND THE DEVELOPMENT OF CORE RESEARCH ETHICS PRINCIPLES

All human beings belong to one human race, however, race, as a social construct, emerged through historical and political processes. As such, racism must be critically examined in research due to its profound material and symbolic consequences, particularly as a social determinant of health that disproportionately harms marginalized and racially minoritized groups such as Black communities.^4^ Black communities refer to people of Sub‐Saharan African descent who experience daily life and self‐identify as Black. Black communities are diverse but share the historical legacy of anti‐Black racism rooted in colonialism, enslavement, exploitation, and segregation. This legacy continues to manifest in contemporary everyday life through interpersonal, institutional, systemic and societal racism. Anti‐Black racism is a pervasive form of racism that specifically targets Black people, which combines ideologies and practices reflected in various singular laws,

**Health scholars are encouraged to familiarize themselves with the history of the Black communities studied, to develop relationships with these communities, and adapt their interpretation of core research ethics principles by taking into account their national and regional contexts.**

policies, customs, stereotypes, and attitudes. These mechanisms produce systemic inequities, leading to Black people's unequal access to symbolic and material resources along with experiences of discrimination, mistreatment, segregation, disenfranchisement, subordination, and dehumanization. We recognize that Black diasporic communities—referring to Black populations located outside of Africa—are diverse in their configurations, worldviews, histories, cultures, national context, and contemporary challenges. We also note that each Black community is not monolithic, and that individual experiences will be shaped by social class, gender, disability status, age, sexual orientation, national origin, migration status, language, religion, and culture. This multilayered and heterogenous understanding of Black experiences informed the panelists’ reflections during the symposium.

The historical legacy of harmful research contributed to the development of the core principles of research ethics as we know them today. Unfortunately, racism has influenced the course of biomedical research and led to human rights violations. During World War II, physicians and scientists working for the Nazi regime injured and murdered concentration camp prisoners, notably Jewish prisoners and children while conducting unethical medical experiments.^5^ The criminal, inhumane, and antisemitic nature of these experiments came to light during the Nuremberg trial of Nazi leaders and officers.^6^ This trial spurred the creation of the Nuremberg Code and is now known as a foundational blueprint in the history of ethical principles in medical research.^7^

Before World War II, in 1932, the Tuskegee experiment was initiated in Tuskegee, Alabama, with 600 African‐American men, 399 of which had syphilis.^8^ In 1943, when penicillin was known and readily available to cure syphilis, researchers failed to offer these Black men penicillin because they prioritized observing the evolution of syphilis in its later stages. In 1972, the lack of concern for the welfare of the participants became public and ultimately led to a settlement for the injured parties or family members in 1974, followed by a public apology from President Clinton in 1997. The Tuskegee experiment, a blatant consequence and example of scientific anti‐Black racism, certainly influenced the U.S. National Commission for the Protection of Human Subjects of Biomedical and Behavioral Research (1979),^9^ whose members described ethical principles to protect human subjects in *The Belmont Report*. Both the Nuremberg Code and *The Belmont Report* continue to influence the advancements and interpretations of international guidelines for health and medical research.^10^

It is important to note that other less known historical events influence the relationship of Black people with biomedical and public health research.^11^ For instance, since the 1950s, Henrietta Lacks's cervical cancer cells, the HeLa cell line, have been used in numerous biomedical studies (e.g., polio, cancer, Ebola disease) without her informed consent.^12^ Similarly, the Holmesburg Prison experiments in Pennsylvania (1950‐1970) subjected mostly Black inmates to dermatological, pharmaceutical, and chemical tests without proper consent. They were exposed to toxic substances and radioactive compounds, often resulting in severe and lasting health consequences.^13^ Additionally, James Marion Sims, often referred to as the “father of modern gynecology”^14^ conducted surgical experiments in the U.S. on enslaved Black women without anesthesia, justifying these inhumane procedures under the false belief that Black women had a higher pain tolerance than white women.^15^ While Canada's history of medical experimentation on Black bodies is less documented, historians reveal that Canada's colonial legacy similarly dehumanized enslaved Black people, making them vulnerable to exploitation, including likely medical interventions that prioritized utility over care.^16^ These cases exemplify the failure to uphold core research ethics principles of respect for persons, beneficence, justice, and informed consent, and how Black bodies have been exploited for research.^17^

In addition to the history of unethical health research, contemporary health disparities and experiences of racial discrimination in health care services fuel the distrust and apprehension of many Black people toward health research and systems in Canada, the U.S., and globally.^18^ This distrust was further amplified during the Covid‐19 pandemic when two French doctors suggested first testing potential treatments in Africa, reigniting historical memories of Black populations being treated as experimental subjects without consent.^19^ Such comments reflect a mindset that devalues Black lives and perpetuates the exploitation of marginalized communities under the guise of medical advancement. Historical cases and ongoing challenges demonstrate the necessity of remaining vigilant and continuing to reflect on what constitutes ethical research practices across contexts. Scholars’ commitment to avoiding a repetition of past unethical practices as they attempt to redress the historical harm health researchers have inflicted on minoritized populations requires a deliberate and frequent engagement with core research ethics principles.^20^

## RESPECT FOR PERSONS AND COMMUNITIES

The first core ethics principle involves obtaining an individual's free, informed, and ongoing consent prior to the use of their data or biological material or prior to their direct participation in a research project. This principle upholds accountability and transparency about the research process while respecting the individual's autonomy and judgment.^21^ In fact, institutional review boards, also known as research ethics boards, require that researchers present meticulous and accurate descriptions of their research project to genuinely secure free, informed, and ongoing consent from each individual participant. Some scholars suggest that to conduct ethical, community‐based, engaged research, it is paramount to respect individuals and affected communities.^22^ We contend that the principle of respect for persons should more explicitly delve into considerations about health researchers’ interactions with historically marginalized communities such as those from the Black diaspora.

During the symposium, several panelists suggested that respect for communities include an examination of the theoretical concepts used to investigate health in Black populations. Theory influences the research project because it informs the research questions asked, the research design selected, the process of data collection, data analysis, and dissemination. We recognize that reframing theoretical frameworks can be a challenge, especially when funding agencies often launch grant competitions about topics that center individual behaviors rather than structural and social determinants of health.^23^ Yet, conceptual frameworks that emphasize “deficit” often lead researchers to disregard the leverage of existing Black communities’ cultural capital while neglecting the analysis of health systems and structures. For instance, Jessica Y. Breland and Michael V. Stanton recommend that health researchers name anti‐Black racism as a structural factor in their theoretical framework and do not focus solely on individual factors and behaviors that are often emphasized, even in socioecological models.^24^ Across disciplines, some theoretical frameworks chosen to analyze social problems reinforce stereotypes and the stigmatization of Black people, while neglecting to shed light on structural factors that contribute to current and pressing challenges. Subsequently, these theoretical selections can lead to short‐lived and ineffectual solutions. It has been suggested that without an acknowledgment of intersectionality, health research and interventions designed to address anti‐Black racism are likely to miss the mark.^25^ Intersectionality is defined as “the critical insight that race, class, gender, sexuality, ethnicity, nation, ability, and age operate not as unitary, mutually exclusive entities, but as reciprocally constructing phenomena that in turn shape complex social inequalities.”^26^ In a context where Black communities are diverse and constantly changing, it is part of the preliminary design work to take into account intersectionality and the sociopolitical history of the Black population studied to write theoretical frameworks that comprehensively advance health equity with a focus on structural barriers.

As it is essential to examine the structural inequities in health systems that contribute to gaps between policies and practices,^27^ echoing other scholars, the symposium panelists encouraged analyses that go beyond a mere examination of low socio‐economic status or migration status because they are not substitutes nor synonyms for anti‐Black racism.^28^ Pushing these boundaries carries the potential of addressing the deprivations experienced by Black community members while producing effective and impactful policy and practice recommendations.^29^ Furthermore, minoritized communities’ resources are often overlooked and devalued, such as the expertise of researchers from these communities. Indeed, several scholars suggest that there is a need to advocate for and elevate Black scholars in health equity research, especially when in the U.S., only a small number of Black scholars receive major funding from the NIH to conduct health equity research compared to white scholars.^30^ To address this discrepancy, health research teams should consider relinquishing power to Black scholars within their team by assigning Black scholars as co‐principal investigators rather than co‐investigators.^31^ Health researchers must also consider the value of centering Black community wealth which consists of “developed, learned and shared cultural capital that is acquired through interaction and social action across generations.”^32^ A concrete way of recognizing Black community wealth is illustrated through the integration of peer research associates in a national study about vaccine hesitancy among Black Canadians.^33^ Peer research associates are Black community members who hold a bachelor's degree in an area relevant to a research project and who are trained and supervised by the research team to raise awareness, engage, and recruit participants in Black communities. Researchers should also consider incorporating epistemological approaches and ways of producing knowledge that the community values or practices.^34^ For instance, scholars have suggested that focus groups can become counterspaces that support and uphold Black women and girls’ agency^35^ and participatory action research holds the potential of improving the health of Black women who reside in rural and remote areas.^36^ Respect for persons and communities also implies that the collected data does not remain confined within academic and medical centers’ walls. It must be returned to the community with the goal of fostering practical solutions and benefits. Given the history of exploitative research in Black communities such as the Tuskegee experiment, scholars may face suspicion and skepticism toward research.^37^ In this context, symposium panelists highlighted that researchers must be ready to answer participants’ questions honestly regarding the use of data, and its transformative potential. For instance, Herman shared how, as part of the Seeding Resilience community coalition in Buffalo, N.Y., he supported the preparation of not‐for‐profit organizations’ federal grant applications to further the goal of food security in Buffalo.^38^ He also underscored that, when possible, scholars should consider working with existing community organizations that are already working toward long‐term health equity research goals (e.g., food security) to optimize our research outputs and these organizations’ initiatives. Dr. Herman's example demonstrates the importance of thinking about the tangible long‐term impact or effects that health research can have for individuals as well as communities. This supports other scholars’ recommendation that researchers engage in multilevel intervention research to affect community members, institutions, or systems to increase the potential impact of health research outputs.^39^

## CONCERN FOR WELFARE AND BENEFICENCE THROUGH COMMUNITY INVOLVEMENT

Concern for welfare, also known as beneficence, refers to the obligation of researchers to uphold the welfare of participants, to minimize participants’ exposure to potential risks and harm throughout the research process, and to inform participants about the potential risks and benefits of their involvement.^40^ Knowledge production can certainly yield benefits, but there are instances where individuals and groups are harmed. Today, researchers are systematically trained to uphold a concern for participants’ welfare to avoid actions that could result in a disregard for human dignity and life. We suggest that health researchers can broaden their interpretation and application of beneficence by encouraging and ensuring community involvement to avoid harm and by creating spaces where Black community members themselves determine how the research process can be beneficial to them.

To uphold the principle of concern for welfare, it is wise to involve the community in the early stages of a research project to minimize risks and optimize the benefits of the research project. Panelists highlighted that before engaging community members, it is important to build relationships to establish trust. This observation coincides with conclusions made by numerous scholars who have conducted research with Black populations who highlight the necessity of taking time to build relationships and trust throughout the research process.^41^ Sadie Goddard‐Durant and colleagues^42^ explain how their first meeting with an Afrocentric community‐based organization was exploratory in nature and consisted of acknowledging the needs of this organization rather than imposing a particular research protocol. The organization's director was interested in a study about young Black mothers, but insisted on the importance of reciprocity, transparency, and relationship building.^43^ The dialogues and interactions in these relationships enable health researchers to anticipate and minimize risks while also uncovering how the research project can yield tangible benefits for the participants’ communities. During the symposium, Salami explained that when she conducted a participatory‐action research project in Alberta about the mental health of Black youth, she approached them to inquire how the research team could support them. These youth oriented the research project in a direction that served their aspirations for the formation of a collective identity that shaped the design of research questions about emerging intergenerational and family issues, and resulted in the creation of tangible initiatives to improve the mental health of Black youth.^44^

Matthews emphasized the importance of teaching cultural humility to future health practitioners and researchers that involves a focus on developing openness and humility in the face of constantly changing cultures while avoiding the essentialization of groups’ cultural practices. As Lekas and colleagues point out, “Cultural humility refers to *an orientation towards caring* for one's patients that is based on: self‐reflexivity and assessment, appreciation of patients’ expertise on the social and cultural context of their lives, openness to establishing power‐balanced relationships with patients, and a lifelong dedication to learning.”^45^ Essential components of health education and research methods’ courses could include understanding the history of medical research abuses^46^ and integrating critical and intersectional frameworks.^47^

Health researchers can also teach their students how they can incorporate community involvement while fostering cultural humility when they conduct research to produce benefits for participants’ communities during the research process. For instance, asking one or several community groups to provide input regarding the direction of a research project increases the likelihood that the process and the outputs of an inquiry will be beneficial for the communities concerned, and instrumental for health equity. Students learning to engage with communities in this manner can develop essential skills in communication, empathy, and reciprocity. They gain firsthand experience in conducting research that is not only scientifically rigorous but socially and ethically responsible, while strengthening fair and meaningful distribution of research benefits. This collaborative approach not only enhances the relevance and applicability of the research but also promotes mutual respect and trust between researchers and community members.^48^

Another way to uphold beneficence mentioned by the members of the panel was the importance of building capacity among Black postsecondary students and community members to conduct research throughout the different phases of an inquiry. For instance, during a participatory‐action research project, Salami trained Black youth to partake in the different phases of the research project and to broaden their knowledge about mental health.^49^ We also suggest that scholars should include Black undergraduate and graduate students in their research teams to expand their professional development opportunities.^50^ Finally, we contend that concern for welfare through community involvement is not a one‐time commitment but an ongoing responsibility that requires researchers to prioritize long‐term impact and sustainability. Moving beyond extractive practices where data is collected and communities are abandoned, researchers must build lasting partnerships that extend well beyond the project timeline. This includes translating findings into actionable, culturally responsive health interventions, such as programs addressing chronic illness or mental health disparities and continually monitoring their long‐term effectiveness. Accountability is key and can be maintained through regular follow‐up, feedback loops, and accessible dissemination of results that empower communities to guide future research priorities. By ensuring these opportunities, health scholars will strengthen the ability of historically excluded and marginalized communities to better grasp the research process, meaningfully contribute to shaping relevant studies, and empower themselves to advance health equity.

## JUSTICE THROUGH INNOVATIVE OUTREACH TO PROMOTE PARTICIPATION

The principle of justice refers to the obligation to give a chance to all to partake in research while treating each individual fairly and equitably without overburdening a group with harm or denying a group the benefit of the knowledge generated from inquiries.^51^ Ruqaiijah Yearby^52^ suggests that the justice principle should include a human development approach that “requires researchers to provide a benefit to the population from which the research subjects originated that alleviates some of the populations’ underlying problems.” This human development approach overlaps with concern for welfare and justice principles. Yet, the benefits of social science research on hard‐to‐reach populations often outweigh the potential risk.^53^ This goal that research benefits a population studied by generating research outputs that alleviate their problems can apply to the health sciences. This can be accomplished by addressing the structural barriers of hidden and underrepresented populations through the research process, without overburdening these populations. We recognize that the meaningful research outputs produced by health researchers to guide policy development may have a limited reach. Scholars must reflect on and plan how they can effectively reach stakeholders and affect policy changes that lead to meaningful impact in the lives of marginalized underrepresented populations. Yet, even before a dissemination plan involving engagement with policy‐makers is in place, the challenge remains to intentionally recruit hard‐to‐reach populations, especially when there is mistrust because of a history of exploitative research such as with Black people.^54^ We contend that concrete and innovative actions should be undertaken to recruit Black people in biomedical and public health research projects while also upholding the principle of justice grounded in a human development approach.

During the panel discussion, it became apparent that when we recruit participants in Black diasporas, intersectionality is important to gather different sense‐making processes, cultural repertoires and lived experiences across linguistic lines. For instance, in an officially bilingual country such as Canada, to ensure that everyone has the possibility of engaging in health research, it is important to strive to recruit participants from both linguistic groups. For instance, during a research project in Quebec, outreach efforts were made to bring across linguistic lines English‐speaking and French‐speaking partners and participants to reflect the needs and expectations of Black communities.^55^ Scholars can achieve such goals with different levels of success, but the efforts remain essential because perspectives in one linguistic group are not necessarily the same in the other.^56^ For instance, Jude Mary Cénat et al.^57^ found that Black residents of Quebec had a higher proportion of vaccine hesitancy than in other provinces because in general, at the national level, French‐speakers have a higher level of vaccine hesitancy compared to English‐speakers. Moreover, structural barriers are context‐dependent and can be affected by the official language spoken in a specific jurisdiction. Among Black French‐speakers outside of Quebec, their dual minority status is also accompanied with tangible structural barriers as they experience challenges accessing health care services in French, including in the context of HIV treatment.^58^ Specific attention to existing multilingual contexts of Black diasporas upholds the principle of justice by ensuring that one linguistic community is not overburdened with research and that members from all linguistic groups can participate in health research and benefit from the research outputs.

Scholars may resort to creative and relational ways of connecting with prospective participants to ensure that Black communities are not overlooked and benefit from the knowledge produced in health research. To this point, Matthews discussed how Black children and youth are underrepresented or absent in speech pathology studies. As a result, some Black children and youth can be misdiagnosed with some assessment instruments. To address this gap in the Canadian context, Matthews organized workshops in Montreal where she presented information about her area of research while serving free culturally relevant food and providing free childcare services to young Black mothers and their children. During the workshop, Matthews introduced herself, her expertise, and the relevance of her research for the well‐being of Black children. At the end of the workshop, she was able to recruit prospective participants for her study about speech development in Black families. At that point, the attendees had grasped the relevance and the benefits of her research project and expressed interest in participating. This example shows how initiatives that allow prospective participants to better know the researchers and the pertinence of the research project are crucial to give a chance to all to participate. Justice with a human development approach is attainable when relevant research projects inform governmental and other stakeholders’ policy decision‐making but also advance the goals and practices of community groups and organizations that work closely with Black populations and other minoritized groups. Boatswain‐Kyte discussed how as part of a research team conducting community participatory‐action research, she witnessed that several participants expressed a desire to have access to the data collected to achieve their own goals.^59^ She discovered that some Black communities may have more interest in understanding the root causes of their conditions and little interest in comparisons with other groups. While the research team initially aimed to demonstrate the discrepancy between the Black community and other groups to government officials to address policy gaps, not‐for‐profit community organizations valued the use of data more to enhance their work and showed minimal interest in the discrepancy analysis. At the same time, the input of the community members compelled the research team to focus on root causes and underestimated contexts and factors that shape Black disenfranchisement rather than a discrepancy study.^60^ We provide this example not as an indication that discrepancy analysis should be abandoned; on the contrary, discrepancy studies certainly have their place and their purpose in health research. Rather, we hope to demonstrate how community involvement enhances the principle of justice and enables researchers to re‐examine their goals and their research outputs.

## CONCLUSION

The insights from Black scholars during a symposium panel provided the opportunity to grow our understanding of how health research can contribute to Black communities thriving, flourishing, and rejoicing, and not simply surviving. The guidance provided by these researchers points to the significance of respecting individuals and communities, of fulfilling beneficence by tapping into community involvement, and of embracing justice with a human development approach by engaging in innovative outreach to benefit Black diasporic populations in a meaningful way. By doing so, this article proposes to expand the interpretation of the core principles of research ethics when health researchers work with Black people and other historically minoritized communities.

Health scholars are encouraged to familiarize themselves with the history of the Black communities studied, to develop relationships with these communities, and adapt their interpretation of core research ethics principles by taking into account their national and regional contexts. Nevertheless, these principles are not static; they require continuous reflection and adaptation to ensure that research practices remain relevant and just. By tending to this reflection, acknowledging and addressing structural inequities, and fostering meaningful community involvement, health researchers can contribute to the safety, healing, and empowerment of disenfranchised communities, creating favorable conditions for equitable health research.

As behavioral, biomedical, and public health research with a focus on racialization and Black populations continues to grow,^61^ health scholars should prioritize evaluating training programs for health researchers and professionals in conjunction with these expanded core principles to ensure the concurrent growth of sustainable cultural humility and equitable applied research ethics. Other intellectual initiatives could explore how these expanded core principles can be interpreted and adapted when health research takes place in the Global South. Continuing reflections about how anti‐Black racism undermines the conduct of ethical health research with various indigenous Sub‐Saharan African groups would certainly enrich our understanding of the core principles of research ethics and equitable research practices.

## ACKNOWLEDGMENTS

This article draws on a symposium entitled “Designing a Flourishing Future and Researching with Black Communities in Canada” held on November 20, 2023, at York University, and was supported by a Social Sciences and Humanities Research Council (SSHRC) Connection grant, a Robarts Centre for Canadian Studies grant, and a York University LA&PS Research Events Funds grant. We would like to thank the panelists and guests who made this symposium a successful and enriching learning event.
